# Comparative Analysis of the Gut Microbial Composition and Meat Flavor of Two Chicken Breeds in Different Rearing Patterns

**DOI:** 10.1155/2018/4343196

**Published:** 2018-10-16

**Authors:** Jing Sun, Yan Wang, Nianzhen Li, Hang Zhong, Hengyong Xu, Qing Zhu, Yiping Liu

**Affiliations:** ^1^Farm Animal Genetic Resources Exploration and Innovation Key Laboratory of Sichuan Province, Sichuan Agricultural University, Chengdu Campus, Chengdu 611130, China; ^2^Chongqing Academy of Animal Science, Chongqing 402460, China; ^3^Key Laboratory of Pig Industry Science, Ministry of Agriculture, Chongqing 402460, China; ^4^Chongqing Key Laboratory of Pig Industry Sciences, Chongqing 402460, China

## Abstract

The objective of the study is to compare the effects of free-range (FR) and cage-range (CR) breeding on gut microbiota and flavor compounds of Caoke (C) and Partridge Shank chickens (Q). A total of 120 experimental chickens were assigned to FR group and CR group; each group contain both 30 Caoke chickens and 30 Partridge Shank chickens. At 154 d old, 12 chickens of each group were selected and their cecal contents were extracted and examined for the composition of gut microbiota by illumina sequencing of the V3 region of the 16S rDNA genes, and flavor compounds were analyzed through headspace-solid-phase microextraction (HS-SPME) method. The results showed that, except for acids, the amount of flavor substances in the FR group was higher than those in the CR group, especially the content of Hexanal and D-limonene. Meanwhile, the higher concentrations of carbonyls including (E,E)-2,4-decadienal, (E)-2-decenal, (E)-2-octenal, and pentanal were in the FR chicken meat, but the differences in concentrations compared with CR were not significant. High levels of ethyl hexanoate and *β*-ocimene were only detected in FR groups. The* Firmicutes* had the highest proportion of chicken cecal microbiota, whereas the* Fusobacteria* was only detected in the cecal samples of Q chicken in FR group.* Actinobacteria *was more prevalent in FR groups than in CR groups. Meanwhile, in Q chickens, the proportions of Bacteroidetes and Proteobacteria in FR group were higher than those in CR group. Using MG-RAST Subsystem Technology, we found that some genes were associated with the formation of precursors of flavor compounds or with the metabolism and degradation of aromatic compounds. Overall, CR and FR breeding influenced the gut microbiota and flavor compounds, potentially because of the changes in diet and living conditions.

## 1. Introduction

Poultry meat is important in the daily life of people by providing abundant protein, fat, and trace elements. As the standard of living develops, more and more people are interested in free-range and organic meat poultry, because the meat of the outdoor chickens had more protein and n-3 polyunsaturated fatty acid than the indoors chickens [1, 2]. However, the benefits of rearing system on chicken product quality and productivity remain elusive. For example, Almasi et al. and Krwaczyk et al. found that free-range rearing has negative on-slaughter weight but has positive effects on meat quality and egg quality [3, 4], while it has no effect on carcass traits and meat quality in chickens. Therefore, search for alternative to replace traditional breeding has gained interest in animal agriculture.

In recent years, greater attention has been given to gut microbial, due to its important role in intestinal development and metabolic homeostasis [5]. The number of studies has rapidly increased finding that gut microbiota in mammals play important roles in digestion of food, synthesis of vitamins and amino acids [6], development of organs [7, 8], regulation of host physiology [9], immune system modulation, and growth and neurodevelopment [10, 11]. For chicken, there are also extensive evidence that microbial composition is influenced by dietary changes, climate, geographical location, pathogenic infections, and antibiotic administration [12, 13]. For example, Ma et al. found that the cecal microbiota of Tibetan chicken from five typical high-altitude regions have slightly diverged due to exposure to different geographic environments [14]. In our previous research, we also revealed that probiotics treatment had significant effects on the microbial community in the caecum of chicken and improved chicken meat flavor [15].

At present, although literature on the impact of rearing pattern on meat quality and healthy in chicken is extensive [3, 16], information on the effect of rearing pattern on the gut environment and composition of the intestinal microbiome of chickens is lacking. To date, in terms of our knowledge, there were only two or three literature about the effect of rearing system on chicken gut microbiome. For example, in the ceca of Dagu chickens raised in free-range systems, a higher abundance of cecal microbiota associated with functions involved in amino acid and glycan metabolic pathways was observed; meanwhile, higher* Firmicutes/Bacteroidetes* ratio was found [17]. Meanwhile, Chen et al. also reported that free-range rearing systems improve the product quality and microbial richness of chickens [18].

Thus, we hypothesized that variation of the feeding regimen might also be linked with the composition of gut microbes, independent of the host genetics. Thus, in this study, we compared the cecum microbes of Caoke and Qingjiaoma chicken lines under free-range and cage-range feeding regimens using next-generation sequencing of 16s rDNA. Specific research objectives included the following: (i) characterizing the flavor compounds in free-range and cage-range chickens of the same age fed the same diet, (ii) characterizing and comparing the microbiota in free-range and cage-range chickens, and (iii) identifying the factors that affect the contribution of the intestinal microbiota to the development of flavor compounds in free-range chickens.

## 2. Materials and Methods

### 2.1. Ethic Statement

All procedures using experimental animals were approved by the Committee on Experimental Animal Management of the Sichuan Agricultural University, permit number 2014-18, and the animals were treated according to the committee's guidelines.

### 2.2. Animals and Sample Collection

The HuaRong Caoke chicken specialized cooperative (Sichuan, Shimian, China) and the Meishan Wens Company (Sichuan, Meishan, China) provided a total of 180 eggs of Caoke Chicken (C, n=90) and Partridge Shank Chicken (Q, n=90), respectively. All eggs were hatched at the Experimental Poultry Breeding Farm of Sichuan Agricultural University (Sichuan, Ya'an, China), and all birds were reared in an indoor pen until 30 d of age. At 30 d, C (n=60) and Q (n=60) chickens with similar body weight were randomly allocated into two groups (30 C and 30 Q per group) and were raised as cage-range (C-CR and Q-CR) and free-range (C-FR and Q-FR) from October 2011 to March 2012. In the cage raising system in current study, each chicken was in a single cage, and the temperature of the conventional cage system was approximately 20°C, with photoperiod 16:8 h light: dark. The chickens in the FR system were raised in a similar indoor house, but these chickens also had free daytime (from 07:30 to 10:00 and from 15:30 to 18:00) access to a paddock (8×4.5m), despite being kept inside at night. The paddocks were not covered with native grass and other foods, but some gravel, leaves, or insects occurred. All CR and FR chickens were offered the same basal diet and water* ad libitum*. No antibiotic drugs or probiotic products were used throughout the entire feeding trial. The feed nutritive content and routine immunization program are provided in Supplementary Table S1A and S1B.

At 154 d of age, 12 healthy birds of a similar weight were randomly selected from each group. All birds were euthanized by cervical dislocation, and then the bloodletting was performed at the neck, with defeathering in dehairing machine for 30-40 seconds. The carcasses were cleaned using 70% alcohol wipes before the chest and abdominal cavity were opened. Both ends of a midpiece of cecum were ligatured by sterilized cotton thread and then cut and promptly placed on ice. The entire process of collecting intestinal contents with the cutting of cecum samples, in addition to the cutting of the entire pectoralis muscle (PM) on the left side, was performed on a thoroughly cleaned workbench and required less than 30 minutes. The gut content samples were preserved in sterile 15 ml polypropylene centrifuge tubes at -20°C until extraction of bacterial genomic DNA. The muscle tissues were preserved at -20°C until determination of flavor substances.

### 2.3. Headspace-Solid-Phase Microextraction (HS-SPME) Analysis

To determine the composition of meat flavor of the cage-range (CR) and free-range (FR) chickens, the stored PM samples of 12 chickens from each group (CR=6 and FR=6) were placed at 4°C overnight to defrost and then were finely chopped by a meat chopper. A 75 *μ*m Carboxen™/Polydimethylsiloxane (CAR/PDMS) StableFlex fiber (Supelco, Bellefonte, PA, USA) was used for the extraction of meat volatiles. At least 2.5 g of each meat sample with sodium chloride (1-1.5 g) and 8 ml of double distilled water was placed in a 15 ml extraction bottle covered by an aluminum foil seal. The headspace vials were placed at 100°C in a thermostatic water bath for 2 hours, and then the volatiles were extracted by exposing the fiber to the vial headspace for 35 min under continuous agitation and heating at 70°C. After extraction, the fiber was desorbed at 250°C for 5 min in the injection port of a gas chromatography (GC).

### 2.4. Gas Chromatography-Mass Spectrometry Apparatus and Conditions

Volatile compounds were analyzed as described by Wang et al. [19] on a GC-MS 2010 Series system, which was equipped with a DB-5MS capillary column (30 m×0.25 mm ×0.25 *μ*m film thickness) (Shimadzu, Tokyo, Japan). The oven temperature program was as follows: from 36°C, hold 3 min, to 60°C at 5°C/min and then from 60°C to 130°C at 6°C/min and finally from 130°C to 230°C at 10°C/min. The mass spectrometer was operated in the electron impact (EI) ionization mode with electron energy of 70 eV. The chromatographic retention times were 10 min, and the chromatograms and spectra were recorded and processed using Enhanced ChemStation software (Agilent Technologies, Shanghai, China).

The identity of the volatile components in the extracts was assigned by the comparison of their retention indices and MS fragmentation pattern with published libraries. The matching compounds were searched in the NIST05, NIST08, PESTEI_3, and PESTNCI3 mass spectral libraries [20] (Stein 1990). To determine the statistical significance of differences in the volatile flavor compounds between free-range and cage-range chicken breast meats, we first used a one-tailed* t*-test to test the homogeneity of data variance and then used an independent, two-sample one-tailed Student's* t-* test.

### 2.5. DNA Extraction, PCR Amplification, and 16S Illumine Sequencing

Cecum contents were collected from 12 chickens per groups (CR=6 and FR=6) for gut microbiome analyses. Total genome DNA was extracted using a QIAamp-DNA Stool Mini Kit (QIAgen, Hilden, Germany) according to the manufacturer's protocol. The concentration and quality of the extracted genomic DNA were assessed with a Quant-IT™ dsDNA BR Assay Kit (Invitrogen, Carlsbad, CA, USA) and NanoVue Plus™ spectrophotometer (Thermo Scientific, Wilmington, DE, USA). The integrity of the extracted DNA was determined by electrophoresis on a 1% agarose gel. To analyze the microbial populations, the hypervariable V3 region of the bacterial 16S rDNA gene was amplified using the following universal primers: 338F (5′-ACTCCTACGGGAGGCAGC-3′) and 533R (5′-TTACCGCGCCTGCTGGCAC-3′) [21]. Polymerase chain reactions (PCRs) were performed in a 20 *μ*l volume with a HotStarTaq® Plus Master Mix kit (contained HotStarTaq plus DNA polymerase, PCR buffer with 3 mM MgCl_2_, and 400 *μ*M of each dNTP; QIAGEN GmbH, Hilden, Germany), 1 *μ*l (10 ng) of extracted DNA, 0.8 *μ*l of each 5 *μ*M primer and double deionized water. Amplification was conducted in an EasyCycler 96 (Analytik Jena AG, Germany) under the following conditions: initial denaturation at 95°C for 3 min and 30 cycles of 30 s at 95°C, 30 s at 55°C, and 45 s at 72°C, followed by 10 min at 72°C. To avoid false-positives, five independent PCR reactions were performed for each sample with a no-template control. The resulting amplicons were then checked on 1% agarose gels, and purification was performed using a Qubit ® dsDNA BR Assay Kit (Invitrogen, Carlsbad, CA, USA) according to the manufacturer's instructions. All purified products were pooled in an equal ratio for subsequent sequencing using the Illumine HiSeq TM 2000 platform by BGI (Shengzhen, China).

### 2.6. Bioinformatics Analyses

Before bioinformatic analysis, raw paired-end Illumine reads were assigned to each sample based on their unique bar code and then truncated by cutting off the bar code and primer sequences. After initial trimming, we merged the sequence reads using Flash (v1.2.7) (https://ccb.jhu.edu./software/FLASH/) with the criterion that the overlap of the assembled reads must be more than 30 bp without misassembling. Merged fastq files were converted to fasta files and exported into Quantitative Insights into Microbial Ecology (QIIME) software (V1.7.0, http://qiime.org/index.html) to identify sequence reads of individual samples. We also used UCHIME in Mothur (version 1.31.2, http://www.mothur.org/) to identify and remove chimeric sequences. After the above analysis, we obtained high-quality clean tags. Operational taxonomic units (OTUs) were picked using de novo OTU picking protocol with a 97% similarity threshold. For each OTU, a representative sequence was screened and used to assign taxonomic composition using the Greengenes database (bacterial OTUs). Then, rarefaction curves, the Chao1 estimate, ACE estimator, the Simpson index, the Shannon-Wiener index, and beta diversity calculations were performed using QIIME. Unweighted UniFrac distance-metrics analysis was performed using OTUs for each sample. Principal component analysis (PCA) was then performed based on the Fast UniFrac distance metric. Finally, a Ward method was conducted in the pvclust package in R (V.2.9.1) for studying multivariate clustering of cecal samples (http://www.is.titech.ac.jp/~shimo/prog/pvclust/). We used MG-RAST Subsystem analysis for the functional annotation of sequences and generation of normalized heat maps (http://www.mg-rast.org/), and the data were compared using a maximum e-value of 1e-05 and a minimum identity of 60%.

## 3. Results

### 3.1. The Feeding Regimen Affects the Types and Concentrations of Chicken Flavor Compounds

Food aroma is an important attribute that greatly influences consumer acceptability. Therefore, firs, we determined the specific volatile flavor compounds in chicken breast under the two different feeding regimens. A total of 57 and 49 compounds were identified from the chicken breast of C-FR and C-CR, respectively; 51 and 43 compounds were identified from the Q-FR and Q-CR groups, respectively. The corresponding volatile constituents (e.g., carbonyls, hydrocarbons, acids, alcohols, and esters) are described in detail in Table 1. With the exception of acids, the amounts of flavor compounds in the FR group were higher than those in the CR group.

Carbonyls were the most prevalent compounds in chicken breast. Among the carbonyls, (E, E)-2, 4-decadienal and (E)-2-decenal were the most important contributors to the “chicken” flavor. The highest concentrations of these carbonyls were in the FR chicken meat, but the differences in concentrations compared with CR were not significant. The compound 4-ethylbenzaldehyde was only detected in the C-FR samples, which contributes to a sweet, fruity, and bitter almond odor (0.15%±0.0002). The most abundant component in all the samples was hexanal, which was significantly higher in the Q-FR group than in the Q-CR group (28.49% versus 16.58%,* P*=0.017). The FR chickens had higher concentrations of some other carbonyls than those in CR chickens (e.g., (E)-2-octenal and pentanal); significant differences in the carbonyl concentrations were detected between the two QJM groups (Table 1). (E)-2-octenal carbonyl imparts a fatty, fresh-fruity odor and taste, whereas pentanal carbonyl provides a fermented, bready, fruity with berry aroma and taste. Two unsaturated aldehydes, (E, E)-2,4-heptadienal and (E)-2-hexenal, were detected in the two CK groups, but not in the Q-CR group (Table 1).

Among the detected methyl ketones, the C-FR samples had the highest content of 2-heptanone (0.79±0.002%). A high content of heterocyclic 2-pentylfuran was found in the C-FR group (3.33±0.010%,* P*=0.0323). High contents of D-Limonene were detected in all breast samples; the highest content was in the Q-FR chickens (15.19%). D-Limonene has a low odor threshold (10 ppb) with a sweet, citrus aroma and taste. Similarly, for *β*-myrcene, the only hydrocarbon, a high content was found in the Q-FR chickens (3.25±0.004% in Q-FR versus 0.74±0.005% in Q-CR,* P*=0.0018); this compound also has a low odor threshold concentration (13 ppb in water). Additionally, high contents of ethyl hexanoate (4.27±0.025% and 0.38±0.003%) were detected in the two FR groups; this compound has a very low threshold odor value (only 1 ppb). Both of these compounds impart a fruity, tropical mango aroma. Additionally, 2-pentyl butyrate, which is one of the aliphatic esters and imparts a very fruity and ethereal odor, was significantly higher in the Q-FR group than in the Q-CR group (*P*=0.02540). However, in Caoke chickens, 2-pentyl butyrate was detected in the free-range group (0.47%) but not in cage-range group. (Z)-3,7-dimethyl-1,3,6-octatriene, known as *β*-ocimene, imparting a floral, citrus odor and taste was detected in the C-FR and Q-FR chicken meats (0.09±0.0010% and 0.11±0.0002%, respectively). Low levels of oleic acid were detected in the Q and C populations; the highest concentration was detected in Q-FR chicken meat (0.15±0.001%).

### 3.2. Sequence Abundance and Diversity of 16S rDNA Gene

To investigate whether gut microbiota of the same chicken race changed under the different feeding regimens, we performed sequencing of the V3 region of the bacterial 16S rDNA gene for cecal content samples from 6 cage-range and 6 free-range chickens (Caoke and Partridge Shank chickens) on the Illumine MiSeq platform. A total of 2,744,168 raw sequences were generated in this study and deposited in the MG-RAST database (http://metagenomics.anl.gov/) (Supplementary Table S2). After sequence denoising, 1,013,222 high-quality sequences remained. The mean number of sequences per sample was 84,435±1045.935 (s.d.) (Supplementary Table S3). From these sequences, 5505 operational taxonomic units (OTUs) were identified at a 97% sequence similarity level with high threshold identity and with an average of 459 OTUs for each sample. After sampling 24,008 reads, the newly discovered OTUs were reduced, and the rarefaction curves tended to attain the saturation plateau with the sampled read number increasing (Supplement Fig.  S1). Additionally, whether from C or Q chicken, the rarefaction curve showed an increasing trend in CR compared with FR, but the difference was not significant.

The complexity of microbial communities in the guts of C and Q chickens was estimated based on alpha-diversity indices (Chao1 and Shannon indices). Chao1 and ACE were indicators for species abundance, whereas the Shannon and Simpson indices estimated the diversity of gut microbiota. No significant difference was found between CR and FR groups within Caoke and Qingjiaoma for all four indices (Table 2 and Supplementary Fig.  S2).

### 3.3. Comparison of Gut Bacterial Composition between Cage-Range and Free-Range Chickens at the Level of Phylum, Family, and Genus

A taxon-dependent analysis using the Ribosomal Database Project (RDP) classifier was conducted to describe the composition of cecal microbiota associated with different feeding regimens. Sixteen phyla were identified, which included* Bacteroidetes, Firmicutes, Proteobacteria, Fusobacteria, Actinobacteria, Planctomycetes, Chlamydiae, Chlorobi, Ascomycota, Nitrospirae, Tenericutes, Deinococcus-Thermus, Cyanobacteria, Verrucomicrobia, Streptophyta, *and* Spirochaetes*. The four predominant bacterial phyla (*Proteobacteria, Bacteroidetes, Firmicutes, and Actinobacteria*) accounted for more than 99.42% of the total sequences, with separate contributions of 4.64%, 9.95%, 80.86%, and 4.04% for FR chicken and 6.43%, 6.33%, 84.54%, and 1.88% for CR chickens, respectively (Supplementary Fig.  S3). The* Firmicutes *had the highest proportion of chicken cecal microbiota (>70.00% in both chicken breeds), whereas the* Fusobacteria *was only detected in the cecal samples of Q-FR chickens (0.39%).* Actinobacteria *was more prevalent in FR groups (6.09±2.53% for C and 2.00±0.22% for Q) than in CR groups (1.84±1.16% for C and 1.92±0.15% for Q). We also found that* Bacteroidetes* and* Proteobacteria* were different between the FR and CR groups in Caoke chickens (FR<CR, 0.85±0.29% versus 2.79±2.27% and 1.74±1.27% versus 8.33±2.93%, respectively). By contrast, in Partridge Shank chickens, the proportions of* Bacteroidetes* and* Proteobacteria* in FR chickens were higher than those in CR chickens (19.05±2.19% versus 9.88±1.41% and 7.55±6.50% versus 4.54±1.86%, respectively; Table 3).

Although bacterial diversity was not altered dramatically, the increase in relative abundance of* Firmicutes* in the gut microbiota indicated that the different feeding regimen altered the bacterial flora in chickens. For Caoke chickens, the ratio of* Firmicutes/Bacteroidetes* increased significantly in the FR group (107.66±0.67,* P*=0.032) compared with that in the CR group (31.03±5.86). However, in Qingjiaoma chickens, the* Firmicutes/Bacteroidetes* ratio in the CR group (8.35±0.68) was clearly higher than that in the FR group (3.69±1.43), although the difference was not significant (*P*>0.05). We used a nonparametric Mann-Whitney* U* test to assess differences, and the percentage of the phylum* Actinobacteria* was significantly different between the FR and CR groups (*P*=0.0438), whereas the percentages of the phyla* Firmicutes* and* Bacteroidetes* were significantly different between the Caoke and Partridge Shank chicken samples (*P*=0.0032 and 0.0122, respectively).

**Figure 1:**
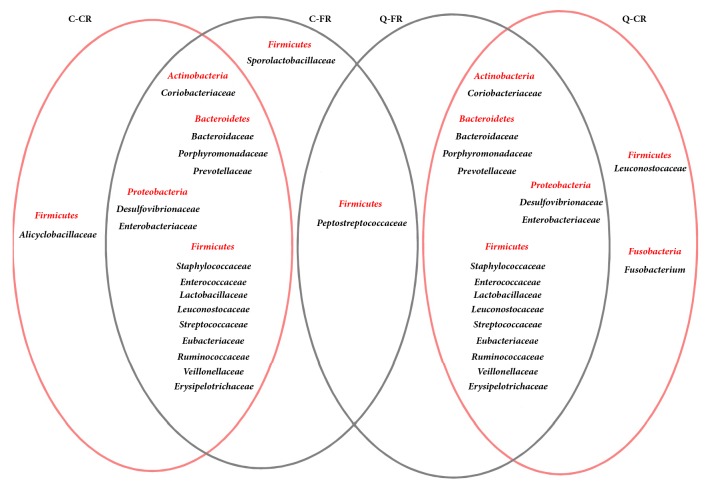
Venn diagrams reveal the different members of the family-level classification in FR and CR chickens.

Notably, at the level of the bacterial “family” (Supplementary Table S4 and Figure 1),* Peptostreptococcaceae* was only found in the two FR groups (0.53±0.34% in C-FR and 1.08±0.23% in Q-FR), whereas* Sporolactobacillaceae* was exclusively in C-FR chickens (2.59±0.60%). These results suggested a potential role of* Peptostreptococcaceae* bacteria in free-range chicken flocks. Alicyclobacillaceae was only found in the C-CR group, and Fusobacteriaceae was only found in the ceca of Q chickens. Moreover, the proportions of the 3 identified core families were significantly higher in the FR group of Caoke chicken than in the CR group, which included Coriobacteriaceae, Staphylococcaceae, and Lachnospiraceae (*P*=0.035, 0.042, and 0.030, respectively). By contrast, the proportion of Lactobacillaceae was lower in the FR group than that in the CR group (*P*=0.003). For Partridge Shank chickens, the proportion of* Peptostreptococcaceae* was significantly higher in the FR group than in the CR group (*P*=0.035).

**Figure 2:**
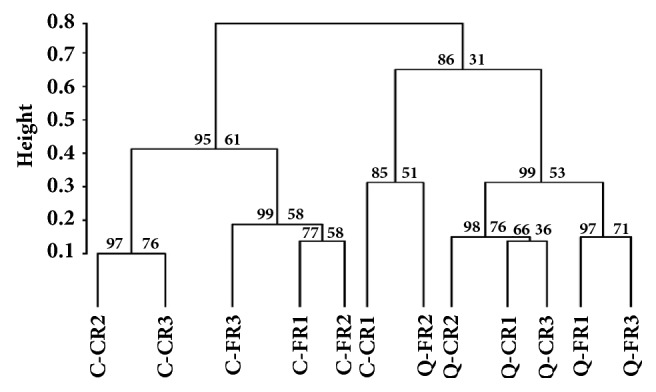
The hierarchical dendrogram of chicken cecal samples. The dendrogram was construct using Ward's method for the samples from the Caoke and the Strain A Partridge Shank chickens with approximately unbiased (AU) and bootstrap probability (BP)* p* values based on the family level of bacterial classification.

**Figure 3:**
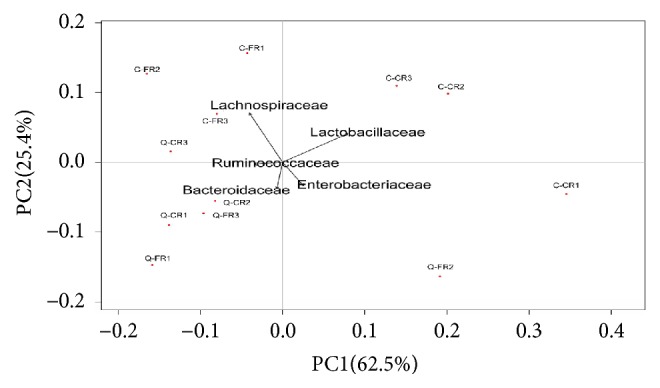
Principal component analysis (PCA) on the family level of bacterial classification in chicken cecal microbiota.

We also measured the cluster similarity of bacterial communities in FR and CR samples within breed according to the “family” level of classification using the* pvclust* package (http://www.is.titech.ac.jp/~shimo/prog/pvclust/). A hierarchical dendrogram was generated using Ward's method with the kld_jsd distance metric (Figure 2). Usually, the p value of AU (approximately unbiased) and BP (bootstrap probability) is the main index to judge the accuracy of clustering. Therefore, as shown in Figure 2, we found that the bacteria composition was similarly between C-CR2 and C-CR3 and Q-FR1 and Q-FR3 at the family level (*P*_*AU*_=97,* P*_*BP*_=76 and PAU=97, PBP=71, respectively). Meanwhile, Q-CR2 and subcluster contained Q-CR1 and Q-CR3 also have the same bacteria composition (P_AU_=98, P_BP_=76). In particular, a subcluster contained only samples of C-CR1 and Q-FR2. Abundant* Proteobacteria *were detected from these two samples (23.26% in C-CR1 and 14.4% in Q-FR2). However, an abnormal increase in* Escherichia* in both the C-CR1 (21.45%) and Q-FR2 (6.21%) birds might be the primary contributor to this unusual subcluster, and the high value of beta-diversity (*β* value=0.404) between the C-CR1 and Q-FR2 samples indicated the distinct habitats of the chickens, although the sample size was limited. Meanwhile, upon PCA analysis, we found that the relative proportion of bacteria in microbiota of cecum in free-range and caged chicken was different. The significant difference was observed along PC1 (P=0.034), the difference was attributed to Bacteroidaceae, Enterobacteriaceae, and Ruminococcaceae, but no difference in PC distribution was seen along PC2. The C chicken of two rearing patterns clustered together; the Q chicken also clustered together (Figure 3).

**Figure 4:**
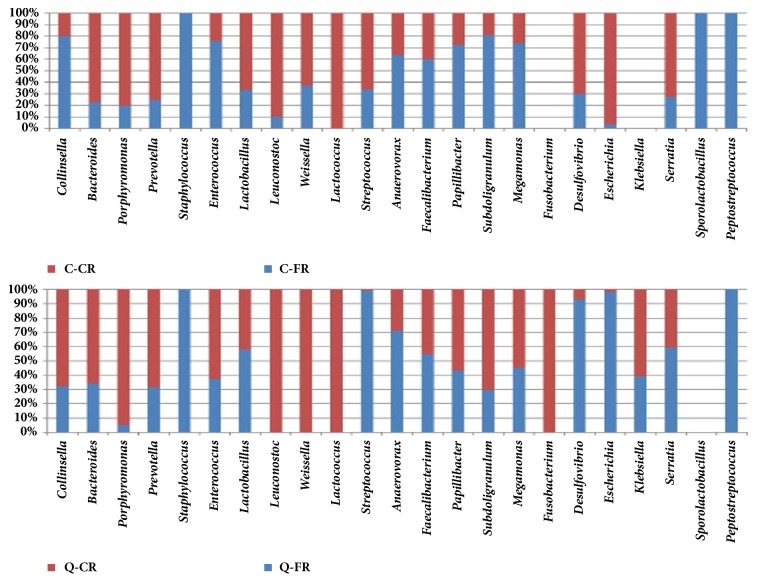
The distribution of bacteria of chicken cecal microbiota at the genus level by full-stacked columns.

At the level of genus as shown in Figure 4, 21 genera were in the cecum of Caoke chicken, but without* Fusobacterium* and* Klebsiella. Staphylococcus* was not found in the CR group (Figure 4 and Supplementary Fig.  S4). The relative abundance of* Lactobacillus*,* Leuconostoc, *and* Lactococcus *was significantly higher in the CR group (45.687%, 0.523%, and 0.116%, respectively) than that in the FR group (22.897%, 0.060%, and 0.003%, respectively;* P*=0.003, 0.036, and 0.026, respectively), whereas the abundance of* Collinsella *decreased significantly in the CR group (*P*=0.028). Similar to Caoke chickens, only the genera* Subdoligranulum* and* Desulfovibrio* had significant differences between the CR and FR groups in Partridge Shank chickens. Some members of the family Enterobacteriaceae are opportunistic pathogens that inhabit the digestive tract of animals; however, no significant differences in the proportions of* Escherichia*,* Klebsiella*, and* Serratia* were detected between the CR and FR groups of the two breeds of chicken. The sample C-CR had an unusually high percentage of* Escherichia* (*P*=0.2164, Supplementary Fig.  S4). An RDP classifier was used to identify the species in the CR and FR samples (Supplementary Table S5). The results showed that gut microbial colonization was feeding-regimen-dependent for the different taxonomic levels of phylum, family, and genus.

### 3.4. The Associations between Gut Microbiota and Gene Functions

To determine whether some genes were associated with the microbial biosynthesis of flavor compounds under different feeding models, we used the MG-RAST Metagenome Annotation using Subsystem Technology (Table 4). Approximately 26 genes regulated lipid or protein metabolism under the different breeding models (Table 4). These genes included 5 enzymes (2-C-methyl-D-erythritol 2,4-cyclodiphosphate synthase; pG1 protein, plastoglobulin 1; RPI, ribose-5-phosphate isomerase; RBKS, ribokinase; 2-dehydropantoate 2-reductase), which were found in all FR and CR samples. Therefore, the gut microbiota in the FR and CR samples contained genes involved in the conversion of dietary carbohydrates into glycolytic/gluconeogenic intermediates that produces NADPH molecules involved in the biosynthesis of fatty acids, particularly 2-dehydropantotae 2-reductase, which participates in the pantothenate biosynthetic pathway and is used in the synthesis of coenzyme A (CoA). In both FR groups, the genes included 2 enzymes (Lipases; Phenylalaine-2-oxo-glutarate transaminase) and 1 protein (LEA domain containing protein). In the C-FR group, genes correlated with microbiota composition were involved in “oxidative phosphorylation” and “glycolysis/gluconeogenesis”, which included aminoacyl-tRNA synthetase, glycerol-3-phosphate dehydrogenase, and cation efflux system protein czcA. Furthermore, in the Q-FR group, two genes that relate to amino acid metabolism, including biotin synthesis and histidinol dehydrogenase, were also identified. Histidine is a precursor of meat flavors and is involved in Maillard reaction products, which can reduce the lipid oxidation in cooked meats and improve their acceptance during storage. These results suggested that alteration of feeding influences the functional processes in the gut.

## 4. Discussion

Our results indicated that the meat flavor and composition and diversity of gut microbiota of chicken were associated with the different housing systems. To our knowledge, in previous research, the focus was on investigating the microbiota of wild and captive individuals of marine mammals [22–24] and pandas [25]; therefore, this study is the first using high-throughput sequencing to examine the gut microbiota in chickens under different housing systems.

Flavor is the most important aspect of meat, often composed of volatile aromatic substances including aldehydes, ketones, esters, phenols, alcohols, organic acids, and alkanes, among others. Therefore, the different proportions of volatile components and the presence of absence of components determine the aroma properties. In our study, 57, 49, 51, and 43 volatile compounds were identified in the C-FR, C-CR, Q-FR, and Q-CR groups, respectively. Aldehydes, including pentanal, hexanal, heptanal, and nonanal, were the most common group of compounds identified in the analyzed samples. Hexanal was the most abundant compound in this study, with an average of 29.48% in the free-range group and 22.5% in the cage-range group. A previous study reported that hexanal primarily originates from linoleic and arachidonic acids [26]. Additionally, Marco et al. also reported that hexanal was the most abundant product of lipid oxidation in meats and might significantly contribute to the overall taste of the product because of the low level of olfactory perception [27]. Moreover, our finding of more aldehydes in the FR group than in the CR group is similar to findings in studies conducted by Cao et al. [28] and Grigorakis et al. [29].

Alcohols were the second most prevalent group of compounds. 1-octen-3-ol was primarily determined in the FR group of chickens, with a low presence in the CR group of chickens. 1-octen-3-ol has a characteristic odor of mushroom and a very low odor threshold [30]. Notably, ethyl hexanoate, an aromatic agent, was only determined in the FR group of chickens and was not detected in the CR group. Additionally, high contents of D-limonene and *β*-myrcene were detected in the FR chickens, and both of these compounds impart a fruity, sweet aroma. Therefore, these differences might be explained by the inherent variability in free-range raising systems, which influenced the concentration of volatile flavor compounds in free-range chicken meat. Other compounds were detected in very low concentrations but most likely have synergistic effects with other compounds that could affect the smell and the taste of chicken meat.

The caecum is a complex ecosystem that includes a highly varied microbiome. In recent years, many studies have used high-throughput sequencing technology to investigate the microbial diversity of the cecum [31–33]. These studies on the microbiome show that* Firmicutes, Proteobacteria,* and* Bacteroidetes* are the three dominant bacterial phyla in the cecal or fecal microbiota of poultry such as chickens [34] and geese [35] and of marine mammals [22]. In this study, for the first time, the effect of housing system on the microbial diversity of chicken cecal samples was studied. At the phylum level,* Proteobacteria*,* Bacteroidetes, Firmicutes,* and* Actinobacteria* were identified as the dominant bacteria in the cecal microbiomes of the CR and FR chickens. However, our results differ from those of Singh et al [36] who reported that* Proteobacteria* was the dominant phylum in chicken fecal samples, followed by* Firmicutes* and* Bacteroidetes*. Notably, in the FR chicken cecum, the dominant phyla from high to low were* Firmicutes*,* Bacteroidetes,* and* Proteobacteria*; however, in the CR chickens, the dominant phyla from high to low were* Firmicutes*,* Proteobacteria,* and* Bacteroidetes*. This difference might be associated with the housing system, because the FR chickens could access the paddock and eat small amounts gravel, leaves, or insects, which are dietary additions that contained higher fiber or mineral content than the feed of CR chickens, leading to the increased abundance of* Bacteroidetes*. This result is consistent with studies on the intestinal microbiome in duck [37] and goose [35] and in the turkey cecum [38]. Similarly, in rabbits and goats,* Bacteroidetes* was the dominant phylum in the intestinal microbiome, and the different composition of nutrient might lead to different effects on biodiversity [39, 40]. Therefore, variations of the dominant phyla of guts may be influenced by diet and environment. Based on our results,* Fusobacteria* and* Klebsiella* were unique to Q chicken and could play a role in cecal digestion, with such a result caused by species-specific differences. However, further work is required to thoroughly understand the effects of breeding mode on the abundance of these bacteria.

We also investigated that the effect of different housing systems on population structure at the genus level. Among the different genera,* Lactobacillus*,* Bacteroides,* and* Faecalibacterium* were the abundant bacteria in the chicken cecum.* Lactobacillus* was one of the differentially abundant taxa that were in much greater proportion than that of other abundant bacteria. Many studies report that* Lactobacillus* is a beneficial commensal for humans and animals, which can improve the gastrointestinal tract and promote the efficient use of nutrients in the host [41]. The relative abundance of* Lactobacillus* in the CR group was significantly higher than that in the FR group, which is finding consistent with that of a study on the red-crowned crane [42]. Because* Lactobacillus* is associated with levels of *β*-xylosidase and *β*-glucosidase in intestinal flora [9], we speculated that the formula diet of the CR group, which contained sugar, led to the greater relative abundance of Lactobacillus in the CR group than in the FR group.

Notably, the abundance of* Bacteroides*,* Porphyromonas,* and* Prevotella *in the CR group was high compared with that in the FR group (Figure 4), which is also similar to the findings of Aguirre et al. [43]. These authors found a high abundance of* Bacteroides* in subjects under a high protein diet but lower counts in subjects under a vegetarian or vegan diet. Similarly, the diet of the CR group primarily contained protein, whereas the diet of the FR group also contained some vegetarian food, in addition to a certain amount of protein. By contrast, the relative abundance of* Anaerovorax* and* Faecalibacterium* was greater in the FR group than that in the CR group.* Faecalibacterium *is the primary bacteria involved in the production of short-chain fatty acids (SCFAs) and although not detected using previous methods, in this study, we detected the rare genus* Faecalibacterium* in all groups.

The unique presence of* Staphylococcus* equorum in the two FR groups (Figure 4) suggested that some airborne or soil-borne biotic pressures affected the intestinal health of the chickens that lived in the free, but more uncontrolled, outdoor environment. The results also revealed that* Peptostreptococcus anaerobius* was prevalent in the two FR groups, but there was no significant difference between C chicken and Q chicken, which expresses the enzyme phenylalaine-2-oxo-glutarate transaminase that catalyzes the conversion of L-phenylalanine and 2-oxoglutarate into phenylpyruvate and L-glutamate [44]. L-glutamate, which is a major flavor-enhancing food component that provides a meaty or savory taste, might be another contributor to the meat flavor of the two FR chicken groups.

Additionally, at the family level, based on our results, many bacteria did not show significant differences between Caoke and Partridge Shank chicken. Franzolin et al. [45] suggested that diet, breed, or geographical location could contribute to the inconsistency of bacterial composition in buffalo. Indeed, for life habit, the Caoke chicken is a type of local chicken that lives at high altitude, has strong resistance to disease, and is primarily free-range raised, whereas the Partridge Shank chicken is a breed similar to the native chicken. Therefore, Caoke chickens use roughage more than Partridge Shank chickens, which might explain some of differential abundance in bacteria between Caoke and Partridge Shank chicken breeds. However, significant differences were observed in bacteria between chickens in different housing systems, particularly for Lactobacillaceae and Lachnospiraceae in Caoke and Partridge Shank chickens, respectively. Our results are consistent with those of some researchers who show that location, age, and environment all play greater roles in shaping the gut microbiota than a trait of the bird itself [46, 47]. Again, these differences might be due to variations in environment, housing system, geographical location, or the different primers that were used.

Based on 16S rDNA gene sequencing, we conducted a comprehensive analysis of the overall composition of the microbial ecosystem in the cecum of Caoke and Partridge Shank chickens with different housing systems. Our data revealed that although some bacteria showed differential abundance between the two different chicken breeds; no significant differences were detected. However, the different housing system treatments had significant effects on the microbial community in the ceca of Caoke and Partridge Shank chickens. Moreover, using the MG-RAST Subsystem Technology, some genes were associated with the formation of precursors of flavor compounds or with the metabolism and degradation of aromatic compounds. These observations provided a better understanding of the effect of housing system on the cecum microbial ecology of Caoke and Partridge Shank chickens.

## Figures and Tables

**Table 1:** Flavor compounds in the FR and CR chicken.

RT^a^ (min)	Component	Relative content (%)			
		C-FR	C-CR	Q-FR	Q-CR
2.083	Ethyl alcohol	0.26 ± 0.004	0.09 ± 0.002	2 ± 0.017	0.04 ± 0.001
3.025	Ethyl acetate	0.14±0.0003^*∗∗*^	0.01 ± 0.0001	0.26 ± 0.001	0.21 ± 0.002
4.467	Pentanal	6.14 ± 0.012	4.73 ± 0.014	4.49 ± 0.016^*∗*^	1.96 ± 0.012
6.633	1-Pentanol	1.34 ± 0.012	1.13 ± 0.011	0.71 ± 0.007	0.45 ± 0.004
7.808	Hexanal	30.46 ± 0.029	28.42 ± 0.035	28.49 ± 0.050^*∗*^	16.58 ± 0.042
9.633	(E)-2-Hexenal	0.24 ± 0.002	0.25 ± 0.001	0.35 ± 0.002	-
10.2	1-Hexanol	0.44 ± 0.0005	0.42 ± 0.004	0.21 ± 0.002	-
10.858	4-Hedroxy-4-methyl-6-phenyltetrahydropyran-2-one	-	-	0.62 ± 0.003	-
10.867	2-Heptanone	0.79 ± 0.002^*∗∗*^	0.15 ± 0.002	-	0.26 ± 0.002
11.275	Heptanal	4.22 ± 0.013	3.27 ± 0.013	1.95 ± 0.004	1.50 ± 0.005
11.658	Oxime-, methoxy-phenyl-	1.04 ± 0.001^*∗*^	0.36 ± 0.004	0.89 ± 0.002	1.20 ± 0.009
11.95	Butyrolactone	0.02 ± 0.0002	0.06 ± 0.0004	0.07 ± 0.0004	0.02 ± 0.0003
13.083	(Z)-2-Heptenal	2.22 ± 0.012	1.21 ± 0.006	0.65 ± 0.005	0.30 ± 0.002
13.233	Benzaldehyde	0.92 ± 0.001	1.05 ± 0.002	1.58 ± 0.005	1.50 ± 0.003
13.517	1-Heptanol	1.19 ± 0.006	0.74 ± 0.005	0.41 ± 0.0007	0.46 ± 0.003
13.717	1-Octen-3-one	0.17 ± 0.001	0.98 ± 0.015	0.11 ± 0.0004	0.08 ± 0.001
13.825	1-Octen-3-ol	2.96 ± 0.007	2.64 ± 0.010	1.67 ± 0.002	0.81 ± 0.008
13.967	2,3-Octanedione	-	-	2.13 ± 0.005^*∗∗*^	0.97 ± 0.002
14.075	2-Pentylfuran	3.33 ± 0.010^*∗∗*^	0.42 ± 0.004	-	-
14.083	*β*-myrcene	4.27 ± 0.025	-	3.25 ± 0.004^*∗*^	0.74 ± 0.005
14.375	Ethyl hexanoate	0.38 ± 0.003	-	0.36 ± 0.170	-
14.525	Octanal	3.50 ± 0.011	1.99 ± 0.017	2.00 ± 0.004	1.78 ± 0.009
14.775	(E,E)-2,4-Heptadienal	0.19 ± 0.002	0.03 ± 0.0003	0.09 ± 0.001	-
15.325	D-limonene	9.77 ± 0.059	5.67 ± 0.054	15.19 ± 0.030^*∗*^	8.67 ± 0.027
15.767	(Z)-3,7-Dimethyl-1,3,6-octatriene	0.09 ± 0.001	-	0.11 ± 0.0002	-
16.125	(E)-2-Octenal	1.77 ± 0.010	0.86 ± 0.007	0.72 ± 0.001^*∗∗*^	0.23 ± 0.002
16.375	(E)-2-Octen-1-ol	0.50 ± 0.001	0.84 ± 0.008	0.17 ± 0.001	0.14 ± 0.0003
16.475	1-Octanol	1.56 ± 0.007	0.93 ± 0.008	0.51 ± 0.002	0.65 ± 0.003
16.817	3-Octanone, 2-methyl-	1.07 ± 0.012	2.10 ± 0.003	-	-
17.133	2-Pentyl butyrate	0.47 ± 0.0007	-	0.22 ± 0.0004^*∗*^	0.09 ± 0.0009
17.425	Nonanal	3.41 ± 0.009	2.28 ± 0.021	1.93 ± 0.007	2.21 ± 0.008
18.333	1-Eicosanol	0.12 ± 0.001	-	-	-
18.708	2-Undecenal	0.07 ± 0.001	0.12 ± 0.001	0.23 ± 0.001	0.20 ± 0.0003
18.892	(E)-2-Nonenal	0.54 ± 0.004	0.29 ± 0.001	0.27 ± 0.001	0.17 ± 0.001
19.017	4-Ethylbenzaldehyde	0.15 ± 0.0002	-	-	-
19.783	Cis-4-decenal	0.09 ± 0.0001	0.05 ± 0.0005	0.05 ± 0.0004	0.05 ± 0.0002
20.067	Decanal	0.22 ± 0.001	0.23 ± 0.001	0.21 ± 0.0004	0.18 ± 0.0006
20.333	(E,E)-2,4-Nonadienal	0.30 ± 0.003	0.09 ± 0.0004	0.05 ± 0.0001	0.04 ± 0.0003
20.775	2-Methyl-1-indanol	0.05 ± 0.0006	0.02±5.77E-05	0.01±5.77E-05	0.03 ± 0.0004
21.067	(S)-2-Cyclohexen-1-one, 2-methyl-5-(1-methylethenyl)-	0.09 ± 0.0005	0.03 ± 0.0002	0.09 ± 0.0002	0.02 ± 0.0004
21.450	(E)-2-Decenal	0.96 ± 0.008	0.28 ± 0.002	0.32 ± 0.001	0.29 ± 0.001
22.442	Pentadecanal	0.04 ± 0.001	0.02 ± 0.0002	0.02 ± 0.002	0.40 ± 0.003
22.692	(E,E)-2,4-Decadienal	0.43 ± 0.003	0.14 ± 0.001	0.30 ± 0.002	0.18 ± 0.0004
23.133	3-Nonen-2-one	0.06 ± 0.0005	0.05 ± 0.0002	0.05 ± 0.0002	0.03 ± 0.0002
23.617	2-Undecenal	0.44 ± 0.004	0.13 ± 0.001	0.23 ± 0.001	0.20 ± 0.0003
24.475	Dodecanal	0.06 ± 0.0001	0.05 ± 0.0003	0.07 ± 0.00	0.07 ± 0.0005
24.848	Benzo[b]thiophene, 2,5-dimethyl-	0.10 ± 0.0007	0.07 ± 0.0006	0.13 ± 0.0008	0.09 ± 0.0005
25.642	1-Tridecanol	0.06 ± 5.77E-05	0.05 ± 0.0003	0.03 ± 0.0002	0.12 ± 0.001
25.783	Heptyl hexanoate	0.02 ± 0.00	0.01 ± 0.0002	0.04 ± 5.77E-05	0.02 ± 0.0002
25.942	1-Tetradecanol	0.02 ± 0.0002	-	0.03 ± 0.0002	-
26.167	Butylated hydroxytoluene	0.15 ± 0.001	0.06 ± 0.0003	-	-
27.683	Heneicosane	1.08 ± 0.099	1.07 ± 0.403	2.35 ± 0.220	5.14 ± 2.11
27.925	Tetradecanal	0.07 ± 0.0002	0.14 ± 0.0005	0.14 ± 0.0008	0.23 ± 0.002
28.367	Decanoic acid decyl ester	0.11 ± 0.001	0.04 ± 0.0002	0.25 ± 0.001	-
30.858	Hexadecanal	0.09 ± 0.0003	0.04 ± 0.0004	0.10 ± 0.001	0.10 ± 0.001
31.442	Diisobutyl phthalate	1.76 ± 0.006	1.77 ± 0.005	5.21 ± 0.006	3.73 ± 0.008
35.142	Oleic acid	0.09 ± 0.0008	0.03 ± 0.0003	0.15 ± 0.001	-

^a^ RT, retention time.

Asterisk (*∗*) represents a significant difference in the amount of volatile compounds between the FR and CR chicken breast meats within the same breed: *∗P *< 0.05; *∗∗P *< 0.01.

**Table 2:** Richness and diversity indexes at an OUT cutoff of 0.03 distance unit.

Sample IDs	Number of OTUs	Alpha Diversity				
		Chao1	ACE	Shannon	Np Shannon	Simpson
C-FR1	294	804	775	2.789	2.807	0.141
C-FR2	349	637	890	3.150	3.172	0.107
C-FR3	477	915	1,383	3.328	3.357	0.100
C-CR1	386	936	1,516	2.243	2.274	0.245
C-CR2	408	652	810	2.758	2.781	0.206
C-CR3	389	623	773	2.685	2.708	0.198
Q-FR1	489	857	1,028	3.961	3.984	0.037
Q-FR2	476	867	1,082	3.206	3.234	0.115
Q-FR3	630	1,191	1,520	3.899	3.929	0.060
Q-CR1	532	1,063	1,281	3.749	3.777	0.054
Q-CR2	544	926	1,145	3.911	3.938	0.049
Q-CR3	531	1,003	1,279	3.701	3.730	0.057

**Table 3:** The average percentage of tag numbers of each phylum in CR and FR chicken cecal microbiota (mean ± S.D.).

Phyla	Caoke (C, %)		Partridge Shank (Q, %)	
	Free-Range	Cage-Range	Free-Range	Cage-Range
	(FR)	(CR)	(FR)	(CR)
*Actinobacteria*	6.09±2.53	1.84±1.16	2.00±0.22	1.92±0.15
*Bacteroidetes*	0.85±0.29	2.79± 2.27	19.05±2.19	9.88±1.41
*Firmicutes*	91.51±2.24	86.57±10.55	70.20±4.21	82.52±2.37
*Proteobacteria*	1.74±1.27	8.33±2.93	7.55±6.50	4.54±1.86
*Fusobacteria*	0.00	0.00	0.39±0.25	0.00

**Table 4:** Functional profile of the chicken microbiota from the MG-RAST subsystem.

Number	Gene functions	Clusters
1	2-C-Methyl-D-erythritol 2,4-cyclodiphosphate synthase	Q-FR, Q-CR; C-FR, C-CR
2	Plastoglobulin-1 protein (pG1 protein), homology to *homo sapiens*	Q-FR, Q-CR; C-FR, C-CR
3	Glucosidase activity in degradation of mucin oligosaccharide chains	Q-FR, Q-CR; C-FR, C-CR
4	Ribose-5-phosphate isomerase	Q-FR, Q-CR; C-FR, C-CR
5	Ribokinase	Q-FR, Q-CR, C-FR, C-CR
6	2-Dehydropantoate 2-reductase	Q-FR, Q-CR; C-FR, C-CR
7	Zinc-containing mannitol-2-dehydrogenase	C-FR, C-CR; Q-CR
8	3,4-Dihydroxy-2-butanone-4-phosphate synthase	Q-CR, Q-FR, C-FR
9	Lytic transglycosylase	C-CR, Q-FR
10	LPXTG-motif cell wall anchor domain protein	Q-FR, Q-CR
11	Utilize citrate for acetoin production	C-CR, Q-CR
12	Utilize cellulose and cellulose derivatives; cellulolytic activity for xylan degradation	C-FR, Q-FR
13	Phenylalaine-2-oxo-glutarate transaminase activity	C-FR, Q-FR
14	Lipases	C-FR, Q-FR
15	LEA domain containing protein	Q-FR, C-FR
16	Putative transcriptional regulator, IcIR family protein	C-FR
17	Aminoacyl-tRNA synthetase	C-FR
18	Glycerol-3-phosphate dehydrogenase [NAD(P)+], anaerobic, A subunit	C-FR
19	Outer membrane adhesion like protein	Q-FR
20	Putative NADPH-dependent glutamate synthase small subunit	Q-FR
21	Methyltransferase involved in both Ubiquinone and menaquinone biosynthesis	C-FR
22	Cation efflux system protein czcA	C-FR
23	Biotin synthesis	Q-FR
24	Histidinol dehydrogenase	Q-FR
25	Polyketide synthase	Q-CR
26	Type IV pilus assembly protein PilB	Q-CR

## Data Availability

All the data generated in this current work are included in the “Result”, and the sequence information of the current study has been uploaded to the MG-RAST database. The specific information is shown in Table S2.

## Supplementary Materials

Supplementary Materials
**S1 Table S1A. Analysis of the total nutrition content in basal feed. S2 Table S1B. The immunization program used in the study. S3 Table S3. Sequencing information summary: number of raw reads, number of clean reads, and percentage of clean data. S5 Table S5. The species assignment for CR and FR populations corresponding to the abundance of bacterial genera exceeding 0.01% found by the RDP classifier. S6 Figure S1. The rarefaction of different cecal samples at the 0.03 distance.** *X-axis represents the number of extracted tags, and Y-axis represents the number of OTUs observed. Dilution curve, 12 colors represent 12 chicken cecal samples of C-CR and C-FR groups in Caoke breed and Q-FR and Q-CR groups in Partridge Shank chicken.* **S7 Figure S2. Comparison of alpha diversity index between FR and CR groups within chicken breeds (Caoke and Partridge Shank).** A. Comparison of chao1 index between FR and CR groups within Caoke/Partridge Shank chicken. B. Comparison of ACE index between FR and CR groups within breed. C. Comparison of Shannon index between FR and CR groups within breed.** S8 Figure S3. Analysis of the 16S rDNA genes of two chicken breeds under different feeding regimens.** Relative abundance (percentage of sequences) of the five most abundant bacterial phyla in each sample among the four groups (C-FR and C-CR groups in the Caoke breed and Q-FR and Q-CR groups in the Partridge Shank chicken).** S9 Figure S4. A histogram showing the number of sequences corresponding to all the genera with the significant difference and the abundance of three genera members in the family Enterobacteriaceae among the FR and CR cecal microbiota.** A one-tailed Student's* t*-test was used to test any differences. Mean values (±SD) are plotted. *∗* P<0.05; *∗∗*P<0.01.
