# Supplementary material for: Comparative Analysis of the Gut Microbial Composition and Meat Flavor of Two Chicken Breeds in Different Rearing Patterns
Source: Biomed Res Int. 2018 Oct 16;2018:4343196. doi: 10.1155/2018/4343196 (PMC6206517; doi:10.1155/2018/4343196)
Supplement: Supplementary Materials — S1 Table S1A. Analysis of the total nutrition content in basal feed. S2 Table S1B. The immunization program used in the study. S3 Table S3. Sequencing information summary: number of raw reads, number of clean reads, and percentage of clean data. S5 Table S5. The species assignment for CR and FR populations corresponding to the abundance of bacterial genera exceeding 0.01% found by the RDP classifier. S6 Figure S1. The rarefaction of different cecal samples at the 0.03 distance. X-axis represents the number of extracted tags, and Y-axis represents the number of OTUs observed. Dilution curve, 12 colors represent 12 chicken cecal samples of C-CR and C-FR groups in Caoke breed and Q-FR and Q-CR groups in Partridge Shank chicken. S7 Figure S2. Comparison of alpha diversity index between FR and CR groups within chicken breeds (Caoke and Partridge Shank). A. Comparison of chao1 index between FR and CR groups within Caoke/Partridge Shank chicken. B. Comparison of ACE index between FR and CR groups within breed. C. Comparison of Shannon index between FR and CR groups within breed. S8 Figure S3. Analysis of the 16S rDNA genes of two chicken breeds under different feeding regimens. Relative abundance (percentage of sequences) of the five most abundant bacterial phyla in each sample among the four groups (C-FR and C-CR groups in the Caoke breed and Q-FR and Q-CR groups in the Partridge Shank chicken). S9 Figure S4. A histogram showing the number of sequences corresponding to all the genera with the significant difference and the abundance of three genera members in the family Enterobacteriaceae among the FR and CR cecal microbiota. A one-tailed Student's t-test was used to test any differences. Mean values (±SD) are plotted. ∗ P<0.05; ∗∗P<0.01. [file 4343196.f1.doc]

**Supporting Informations**

**S1 TableS1A. Analysis of the total nutrition content in basal feed.**

**S2 TableS1B. The immunization program used in the study.**

**S3 Table S2. The sequence information of the chicken cecal microorganism.**

**S4 Table S3. Sequencing information summary: number of raw reads, number of clean reads and percentage of clean data.**

**S5 Table S5. The species assignment for CR and FR populations corresponding to the abundance of bacterial genera exceed 0.01% found by the RDP classifier.**

**S6 Figure S1. The rarefaction of different cecal samples at the 0.03 distance.** X *axis represents the number of extracted tags, and Y axis represents the number of OTUs Observed. Dilution curve, 12 colors represent 12 chicken cecal samples of C-CR and C-FR groups in Caoke breed and Q-FR and Q-CR groups in* *Partridge Shank chickens.*

**S7 Figure S2. Comparison of Alpha diversity index between FR and CR groups within chicken breeds (Caoke and Partridge Shank chickens).** A. Comparison of chao1 index between FR and CR groups within Caoke/Partridge Shank chickens. B.Comparison of ACE index between FR and CR groups within breed. C.Comparison of Shannon index between FR and CR groups within breed.

**S8 Figure S3. Analysis of the 16S rDNA genes of two chicken breeds under different feeding regimens.** Relative abundance (percentage of sequences) of the five most abundant bacterial phyla in each sample among the four groups (C-FR and C-CR groups in the Caoke breed and Q-FR and Q-CR groups in the Partridge Shank chickens).

**S9 Figure S4. A histogram showing the number of sequences corresponding to all the genera with the significant difference and the abundance of three genera members in the family Enterobacteriaceae among the FR and CR cecal microbiota.** A one-tailed student’s t test was used to test any differences. Mean values (± SD) are plotted. * P< 0.05; ** P< 0.01.

**Table S1A . Analysis of the total nutrition content in basal feed.**

| Ingredients | 0-6 week old | 7-17 week old | 18-22 week old |
| --- | --- | --- | --- |
| ^1^Caoke chicken | | | |
| Crude Protein (%) | 20.14 | 18.52 | 17.65 |
| Crude Cellulose (%) | 2.94 | 3.95 | 4.6 |
| Energy (MCal/kg of DM) | 2.93 | 2.89 | 2.85 |
| Protein ratio (g/MJ) | 68.74 | 64.08 | 62.15 |
| Lysine (%) | 1.03 | 0.83 | 0.8 |
| DL-Methionine (%) | 0.45 | 0.42 | 0.45 |
| Calcium carbonate (%) | 0.99 | 0.83 | 0.75 |
| Dicalcium phosphate (%) | 0.8 | 0.75 | 0.63 |
| Available phosphorous (AP, %) | 0.59 | 0.54 | 0.45 |
| Salt (%) | 0.4 | 0.4 | 0.4 |
| Partridge Shank chickens | | | |
| Corn (%) | 58.08 | 66.58 | 72.15 |
| Wheat Bran (%) | 4.21 | 0.00 | 0.00 |
| Puffed soybean (%) | 0.00 | 0.00 | 10.97 |
| Soy bean pulp (%) | 26.21 | 19.23 | 0.00 |
| Rapeseed extraction (%) | 2.79 | 5.26 | 8.15 |
| Dicalcium phosphate (%) | 1.88 | 1.59 | 1.22 |
| Calcium carbonate (%) | 0.91 | 0.83 | 0.80 |
| DL-Methionine (%) | 0.17 | 0.14 | 0.10 |
| ^2^Mineral-vitamin premix (%) | 0.56 | 0.56 | 0.56 |
| Choline (%) | 0.11 | 0.11 | 0.11 |
| Salt (%) | 0.42 | 0.42 | 0.42 |
| Bentonite (%) | 0.32 | 0.42 | 0.43 |
| Mildew preventive (%) | 0.11 | 0.11 | 0.11 |

^1^ All feed used in the whole life of the birds were self-developed by HuaRong Caoke chicken specialized cooperative, Shimian County, Sichuan Province (China).

^2^The mineral-vitamin premix provided the following (per kg of diet): 5,000,000 IU of vitamin A; 200,000 IU of vitamin D3; 5,000 mg of vitamin E; 2,000 mg of vitamin B1; 2,500 mg of vitamin B2; 1,000 mg of vitamin B6; 10 mg of vitamin B12; 10,000 mg of vitamin PP; 8,000 mg of D-pantothenic acid; 25,000 mg of vitamin C; 1,500 mg of vitamin K; 8,000 mg of inositol; 5.13 mg of Fe; 101.1 mg of Cu; 317.8 mg of Mn; 561.4 mg of Zn; 12.6 mg of I; and 36 mg of Se.

**Table S1B. The immunization program used in the study.**

| Age of birds | Name of vaccine | Route |
| --- | --- | --- |
| 1 day | Marek’s vaccine (in Hatchery) | Intramuscular |
| 7 to 9 days | La Sota Newcastle vaccine and Infectious Bronchitis (1st dose) | Intranasal drop + eye drops |
| 13 days | Infectious Bursal Disease Virus (IBDV) vaccine | Drinking water |
| 17 days | Bird Flu vaccine (0.3 ml per bird) | Intramuscular |
| 20 days | Flow pox (1st dose) | Wing web |
| 21 days | La Sota Newcastle + Infectious Bronchitis H120 vaccines | Drinking water |
| 45 days | La Sota Newcastle + Infectious Bronchitis H120 vaccines; Newcastle disease oil-inactivated vaccine (0.3 ml per bird) | Drinking water + Intramuscular |
| 75 days | La Sota Newcastle + Infectious Bronchitis H120 vaccines | Drinking water |
| 85 days | Avian Influenz vaccine | Intramuscular |
| 120 days | La Sota Newcastle + Infectious Bronchitis H120 vaccines | Drinking water |

**Table S2. The sequence information of the chicken cecal microorganism.**

| MG-RAST ID | Sample name | Job link | Project Name |
| --- | --- | --- | --- |
| 4525959.3 | C-CR2 | http://metagenomics.anl.gov/metagenomics.cgi?page=MetagenomeOverview&metagenome=4525959.3 | chicken cecum_A strains |
| 4525960.3 | C-CR3 | http://metagenomics.anl.gov/metagenomics.cgi?page=MetagenomeOverview&metagenome=4525960.3 | chicken cecum_A strains |
| 4525961.3 | C-FR1 | http://metagenomics.anl.gov/metagenomics.cgi?page=MetagenomeOverview&metagenome=4525961.3 | chicken cecum_A strains |
| 4525962.3 | C-FR2 | http://metagenomics.anl.gov/metagenomics.cgi?page=MetagenomeOverview&metagenome=4525962.3 | chicken cecum_A strains |
| 4525963.3 | C-FR3 | http://metagenomics.anl.gov/metagenomics.cgi?page=MetagenomeOverview&metagenome=4525963.3 | chicken cecum_A strains |
| 4525964.3 | Q-CR1 | http://metagenomics.anl.gov/metagenomics.cgi?page=MetagenomeOverview&metagenome=4525964.3 | chicken cecum_A strains |
| 4525965.3 | Q-CR2 | http://metagenomics.anl.gov/metagenomics.cgi?page=MetagenomeOverview&metagenome=4525965.3 | chicken cecum_A strains |
| 4525966.3 | Q-CR3 | http://metagenomics.anl.gov/metagenomics.cgi?page=MetagenomeOverview&metagenome=4525966.3 | chicken cecum_A strains |
| 4525967.3 | Q-FR1 | http://metagenomics.anl.gov/metagenomics.cgi?page=MetagenomeOverview&metagenome=4525967.3 | chicken cecum_A strains |
| 4525968.3 | Q-FR2 | http://metagenomics.anl.gov/metagenomics.cgi?page=MetagenomeOverview&metagenome=4525968.3 | chicken cecum_A strains |
| 4525969.3 | Q-FR3 | http://metagenomics.anl.gov/metagenomics.cgi?page=MetagenomeOverview&metagenome=4525969.3 | chicken cecum_A strains |
| 4526011.3 | C-CR1 | http://metagenomics.anl.gov/metagenomics.cgi?page=MetagenomeOverview&metagenome=4526011.3 | chicken cecum_A strains |

**Table S3. Sequencing information summary: number of raw reads, number of clean reads and percentage of clean data.**

| Sample IDs | Number of raw reads | Clean reads^1^ | Clean data (bp)^2^ | Clean data (%)^3^ |
| --- | --- | --- | --- | --- |
| C-FR1 | 206,624 | 85,112 | 25,533,600 | 41.19 |
| C-FR2 | 192,948 | 85,470 | 25,641,000 | 44.30 |
| C-FR3 | 309,549 | 84,798 | 25,439,400 | 27.39 |
| C-CR1 | 286,097 | 86,100 | 25,830,000 | 30.09 |
| C-CR2 | 155,087 | 84,756 | 25,426,800 | 54.65 |
| C-CR3 | 267,693 | 83,302 | 24,990,600 | 31.12 |
| Q-CR1 | 175,168 | 85,492 | 25,647,600 | 48.81 |
| Q-CR2 | 185,600 | 84,848 | 25,454,400 | 45.72 |
| Q-CR3 | 188,424 | 83,512 | 25,053,600 | 44.32 |
| Q-FR1 | 309,873 | 83,256 | 24,976,800 | 26.87 |
| Q-FR2 | 175,032 | 83,126 | 24,937,800 | 47.49 |
| Q-FR3 | 292,073 | 83,450 | 25,035,000 | 28.57 |

^1^Clean reads: the total number of filtered reads by a set of cleaning steps that involved vector and adaptor removal, the bad quality sequence trimming and short sequence filtering.

^2^Clean data (bp): the total length of clean reads.

^3^Clean data (%): the percentage of clean data to raw data.

**Table S4.** The abundance (mean±S.D.) of bacterial “Family” in the cecal microbiota of FR chickens and CR chickens.

| Family | Abundances (%) | | | |
| --- | --- | --- | --- | --- |
|  | C-FR group | C-CR group | Q-FR group | Q-CR group |
| Coriobacteriaceae | 3.7867±1.5059 | 0.8933± 0.5363 | 0.7333±0.3932 | 0.8133±0.3066 |
| Bacteroidaceae | 0.7100± 0.1108 | 3.7633±3.2223 | 11.0867±0.2886 | 5.4333±2.7209 |
| Porphyromonadaceae | 0.0133± 0.0023 | 0.1033±0.0776 | 0.8300±0.1571 | 1.7067±1.3812 |
| Prevotellaceae | 0.0433± 0.0177 | 0.2367±0.1882 | 3.8867±1.0807 | 1.8600±1.0475 |
| Staphylococcaceae | 0.1133±0.0416 | 0.0000 | 0.7633±0.6153 | 0.0033±0.0005 |
| Enterococcaceae | 0.1567±0.0907 | 0.0567± 0.0152 | 2.2067±1.4521 | 3.7000±3.4001 |
| Lactobacillaceae | 22.8967±5.2380 | 45.6867±3.4750 | 20.3200±3.4419 | 14.8167±3.6744 |
| Leuconostocaceae | 0.0967±0.0750 | 0.5567 ± 0.2886 | 0.0000 | 3.3700±0.9801 |
| Streptococcaceae | 0.2833± 0.0513 | 0.6733± 0.4980 | 2.4667 ± 2.0385 | 0.8267±0.5244 |
| Eubacteriaceae | 0.7867 ± 0.6784 | 0.5067 ± 0.2589 | 0.5700 ± 0.2433 | 0.2567±0.0416 |
| Lachnospiraceae | 40.4567±6.0168 | 22.7100±7.1402 | 19.4367±7.1179 | 29.7233±4.8952 |
| Peptostreptococcaceae | 0.0633 ± 0.0251 | 0.2700 ± 0.1328 | 0.7333±0.2400 | 0.0133±0.0115 |
| Ruminococcaceae | 17.6500±5.2790 | 8.7900 ± 4.6088 | 16.4600±8.4136 | 18.3867±1.9689 |
| Veillonellaceae | 0.2733±0.0355 | 0.2067±0.1755 | 2.1700±1.5384 | 2.2467±1.9633 |
| Erysipelotrichaceae | 4.4267±1.4429 | 2.2167±1.4176 | 1.2867±1.0718 | 2.1600±0.9889 |
| Desulfovibrionaceae | 0.0300 ± 0.0020 | 0.0767±0.0638 | 1.1000±0.7308 | 0.1067±0.0230 |
| Enterobacteriaceae | 0.5300±0.4015 | 7.8700±2.9127 | 6.0533±1.7129 | 4.1133±1.9266 |
| Fusobacteriaceae | - | - | 0.0033±0.0005 | 0.3867±0.0354 |

**Table S5.** The species assignment for CR and FR populations corresponding to the abundance of bacterial genera exceed 0.01% found by the RDP classifier.

| Genus | Species in C-FR | Species in C-CR | Species in Q-FR | Species in Q-CR |
| --- | --- | --- | --- | --- |
| Collinsella | C. aerofaciens | C. aerofaciens | C. aerofaciens | C. aerofaciens |
| Bacteriodes | B. barnesiae | B. barnesiae | B. barnesiae | B. barnesiae |
|  |  | B. caccae | B. plebeius |  |
|  | B. coprocola | B. coprocola | B. coprocola | B. coprocola |
|  | B. coprophilus | B. coprophilus | B. coprophilus |  |
|  | B. dorei |  |  | B. dorei |
|  |  | B. coprosuis | B. coprosuis | B. coprosuis |
|  | B. finegoldii | B. finegoldii | B. helcogenes |  |
|  | B. fragilis | B. fragilis | B. fragilis | B. fragilis |
|  | B. nordii | B. nordi | B. nordi |  |
|  |  |  |  | B. oleiciplenus |
|  | B. salanitronis | B. salanitronis | B. salanitronis | B. salanitronis |
|  | B. sp. 1AL |  | B. sp. 1AL |  |
|  |  | B. ovatus | B. ovatus | B. ovatus |
|  | B. uniformis |  |  |  |
|  | B. vulgatus | B. salyersiae | B. tectus |  |
|  |  | B.thetaiotaomicron | B.thetaiotaomicron | B.thetaiotaomicron |
|  |  | B. uniformis | B. uniformis | B. uniformis |
|  |  | B. vulgatus | B. vulgatus | B. vulgatus |
| Porphyromonas |  |  |  | P.catoniae |
|  | P. crevioricanis |  |  | P. crevioricanis |
|  |  |  |  | P. endodontalis |
|  |  |  |  | P. uenonis |
| Prevotella | P. denticola | P. denticola | P. denticola |  |
|  |  | P. brevis | P. loescheii |  |
|  |  |  | P. melaninogenica |  |
|  | P. multiformis | P. multiformis | P. multiformis |  |
|  |  |  | P. oralis |  |
|  | P. oris |  | P. oris |  |
|  |  |  | P. pallens | P. ruminicola |
|  | P. veroralis | P. veroralis | P. veroralis | P. veroralis |
| Staphylococcus | S. equorum |  | S. equorum |  |
|  |  |  | S. aureus | S. aureus |
|  |  |  | S. epidermidis |  |
|  | S. kloosii |  | S. kloosii |  |
|  |  |  | S. piscifermentans |  |
|  | S. saprophyticus |  | S. saprophyticus | S. saprophyticus |
|  |  |  | S. sciuri |  |
|  |  |  | S. succinus |  |
|  | S. vitulinus |  | S. vitulinus |  |
| Enterococcus | E. avium | E. avium | E. avium | E. avium |
|  |  |  |  | E. asini |
|  | E. canintestini |  |  | E. canintestini |
|  | E. casseliflavus | E. casseliflavus | E. casseliflavus | E. casseliflavus |
|  |  |  |  | E. cecorum |
|  | E. columbae |  | E. columbae | E. columbae |
|  |  |  |  | E. devriesei |
|  | E. durans |  |  | E. durans |
|  | E. faecium |  | E. faecium | E. faecalis |
|  |  |  |  | E. hermanniensis |
|  |  | E. italicus |  | E. italicus |
|  | E. malodoratus |  |  | E. mundtii |
| Lactobacillus | L. acidophilus | L. acidophilus | L. acidophilus | L. acidophilus |
|  |  |  | L. acidipiscis | L. acidipiscis |
|  | L. agilis |  | L. agilis | L. agilis |
|  |  | L. backi | L. backi | L. backi |
|  | L. amylovorus | L. brevis | L. amylovorus | L. amylovorus |
|  | L. animalis | L. animalis | L. animalis |  |
|  | L. aviarius | L. aviarius | L. aviarius | L. aviarius |
|  |  |  |  | L. bifermentans |
|  |  |  |  | L. brevis |
|  | L. cacaonum |  | L. cacaonum | L. cacaonum |
|  |  | L. coryniformis | L. coryniformis | L. coryniformis |
|  | L. casei | L. casei | L. casei | L. casei |
|  | L. coleohominis | L. coleohominis | L. coleohominis | L. coleohominis |
|  | L. crispatus | L. crispatus |  |  |
|  | L. curvatus |  | L gastricus | L. curvatus |
|  |  | L. gasseri |  | L. gasseri |
|  | L. delbrueckii | L. mindensis |  |  |
|  | L. dextrinicus |  |  | L. dextrinicus |
|  | L. equi | L. equi | L. equi | L. equi |
|  | L. fermentum |  | L. fermentum | L. fermentum |
|  |  |  | L. hamsteri | L. mindensis |
|  | L. helveticus | L. helveticus | L. helveticus | L. helveticus |
|  | L. johnsonii | L. johnsonii | L. johnsonii | L. johnsonii |
|  |  |  |  | L. ingluviei |
|  | L. mali |  |  | L. mali |
|  |  |  | L. murinus | L. manihotivorans |
|  | L. oris | L. oris | L. oris | L. oris |
|  |  |  | L.paracasei | L. pantheris |
|  |  |  |  | L. parabrevis |
|  | L. paracollinoides |  | L. paracollinoides | L. paracollinoides |
|  |  | L. plantarum |  | L. plantarum |
|  |  |  |  | L. paralimentarius |
|  | L. perolens | L. saerimneri | L. saerimneri | L. sp. DCY51 |
|  | L. pontis | L. pontis | L. pontis | L. spicheri |
|  | L. reuteri | L. reuteri | L. reuteri | L. reuteri |
|  | L. rossiae | L. rossiae | L. rossiae | L. rossiae |
|  | L. sakei | L. sakei | L. sakei | L. sakei |
|  | L. salivarius | L. salivarius | L. salivarius | L. salivarius |
|  | L. secaliphilus | L. secaliphilus | L. secaliphilus |  |
|  | L. sp. DCY51 | L. satsumensis |  | L. sanfranciscensis |
|  |  |  |  | L. sharpeae |
|  | L. vaccinostercus | L. vaccinostercus |  | L. vaccinostercus |
|  | L. vaginalis | L. vaginalis |  |  |
| Leuconostoc | L. citreum | L. citreum |  | L. citreum |
|  |  | L. gasicomitatum |  | L. inhae |
|  |  | L. lactis |  | L. lactis |
|  | L.pseudomesenteroides | L.pseudomesenteroides |  | L.pseudomesenteroides |
| Weissella |  |  |  | W. cibaria |
|  | W. paramesenteroides | W. ghanensis |  | W. ghanensis |
|  | W. hellenica | W. hellenica |  | W. hellenica |
|  | W. minor | W. koreensis |  | W. minor |
| Streptococcus | S. gallolyticus | S. gallolyticus | S. gallolyticus |  |
|  | S. sobrinus | S. phocae | S. halichoeri | S. parauberis |
|  |  | S. anginosus | S. pyogenes | S. sobrinus |
|  | uncultured Streptococcus sp. | uncultured Streptococcus sp. | uncultured Streptococcus sp. | uncultured Streptococcus sp. |
| Lactococcus |  |  |  | L. garvieae |
|  | L. lactis | L. lactis |  | L. lactis |
|  | L. raffinolactis | L. raffinolactis |  | L. raffinolactis |
| Faecalibacterium | F.prausnitzii | F. prausnitzii | F. prausnitzii | F. prausnitzii |
| Fusobacterium |  |  |  | F. varium |
| Escherichia | E.coil | E.coil | E.coil | E.coil |
| Serratia | S. liquefaciens | S. liquefaciens | S. liquefaciens | S. liquefaciens |
|  | S. marcescens | S. marcescens | S. marcescens |  |
|  | uncultured Serratia sp. |  |  |  |
| Klebsiella |  |  | uncultured Klebsiella sp. | uncultured Klebsiella sp. |
|  |  |  |  | K. pneumoniae |
| Desulfovibrio |  |  | D. alaskensis |  |
|  |  | D. alkalitolerans | D. alkalitolerans |  |
|  | uncultured Desulfovibrio sp. | uncultured Desulfovibrio sp | uncultured Desulfovibrio sp. |  |
| Enterorhabdus | E. caecimuris |  | E. caecimuris |  |
|  | E. mucosicola |  | E. mucosicola |  |
| Eggerthella | E. lenta | E. lenta | E. lenta | E. lenta |
| Peptostreptococcus | P. anaerobius |  | P. anaerobius |  |
| Sporolactobacillus | S. kofuensis |  |  |  |
| Alicyclobacillus |  | A.tolerans |  |  |
| Corynebacterium | C. glutamicum | C. glutamicum | C.glutamicum |  |
|  | C. ammoniagenes |  | C. ammoniagenes |  |
|  | C. freneyi |  | C. capitovis |  |
|  |  | C. flavescens | C. flavescens |  |
|  |  | C. sp. NML93-0612 | C. heansenii |  |
|  |  |  | C. pseudogenitalium |  |
|  |  |  | C. singlulare |  |
|  |  |  | C. sp. |  |
|  |  | C. variabile | C. variabile |  |
|  |  |  | C. xerosis |  |


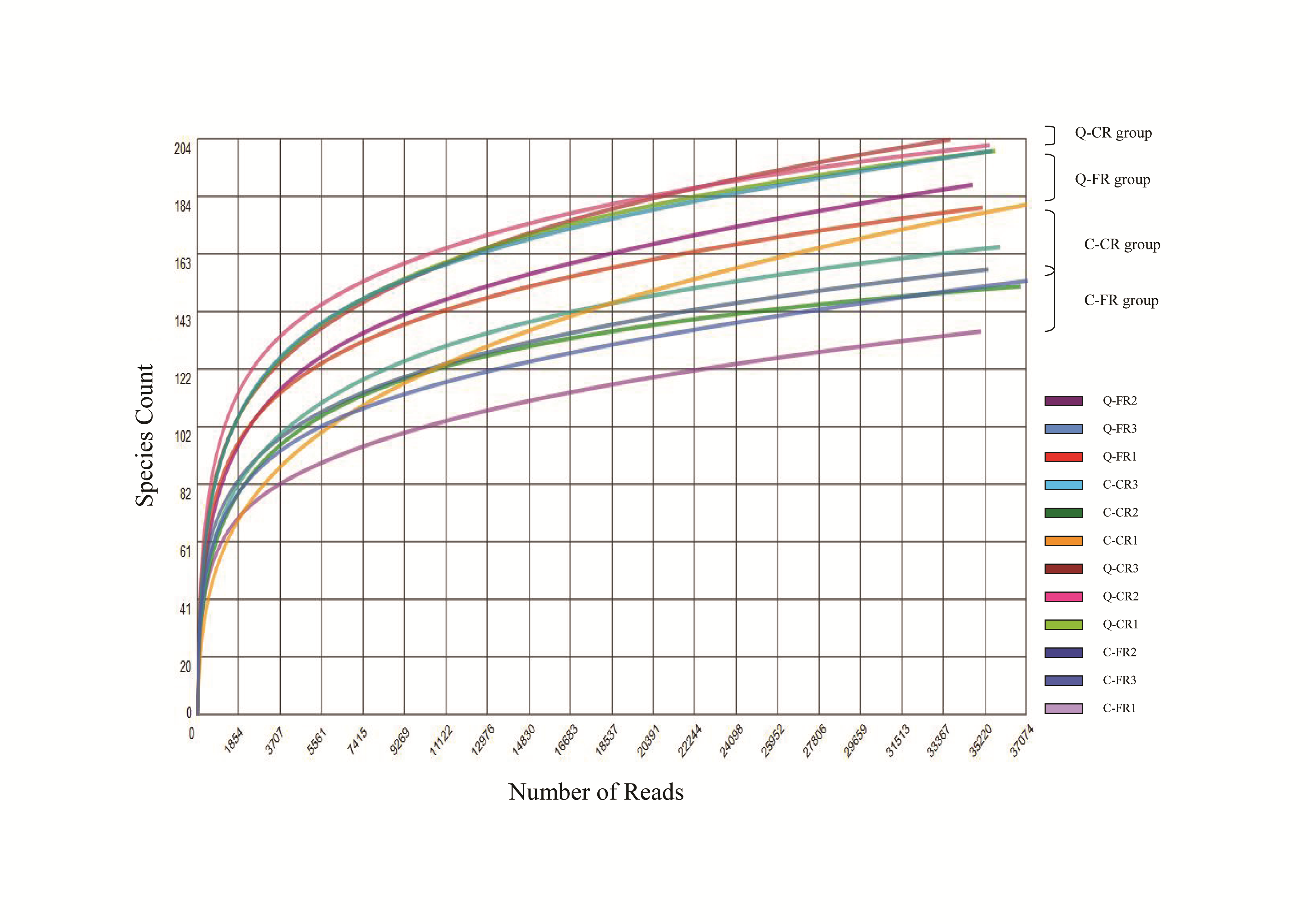


**Figure. S1**

**
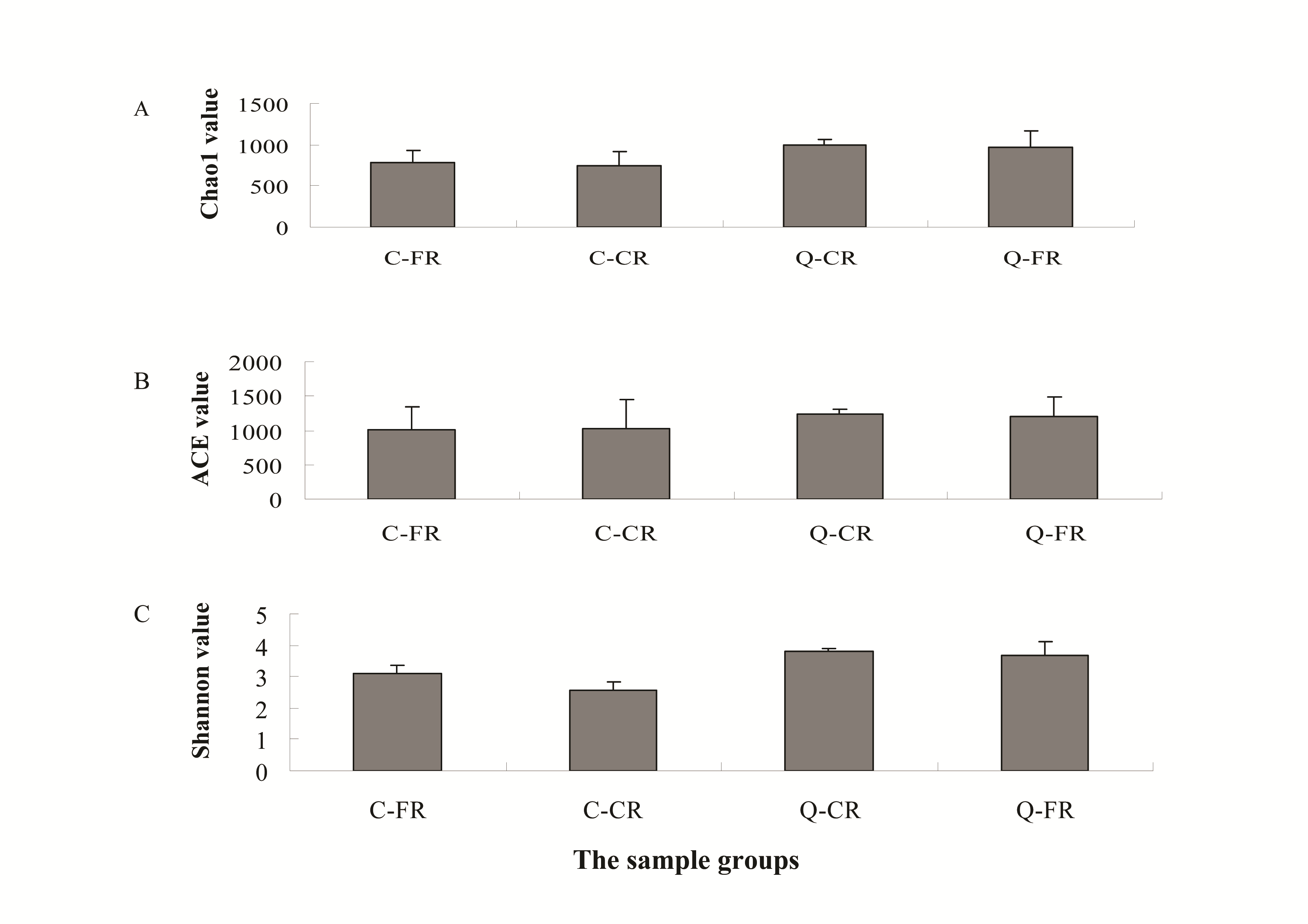
**

**Figure. S2**

**
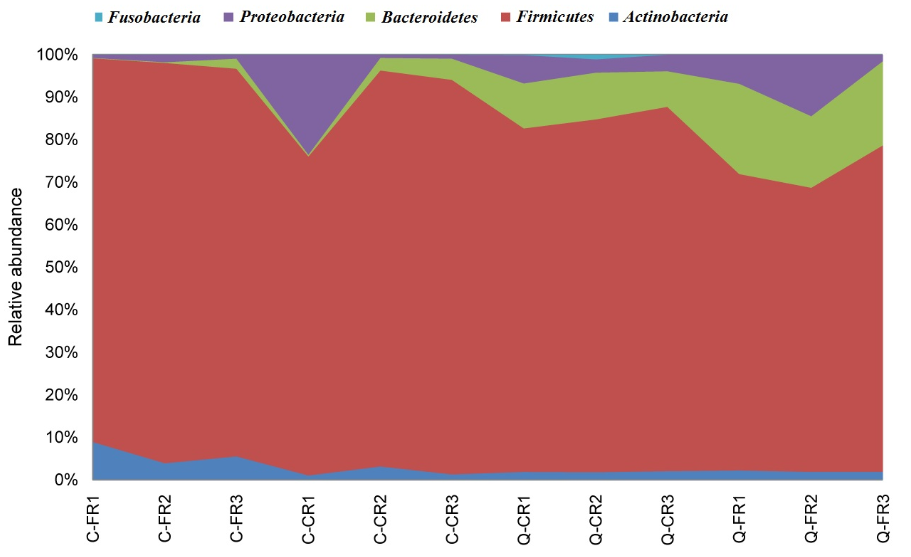
**

**Figure. S3**


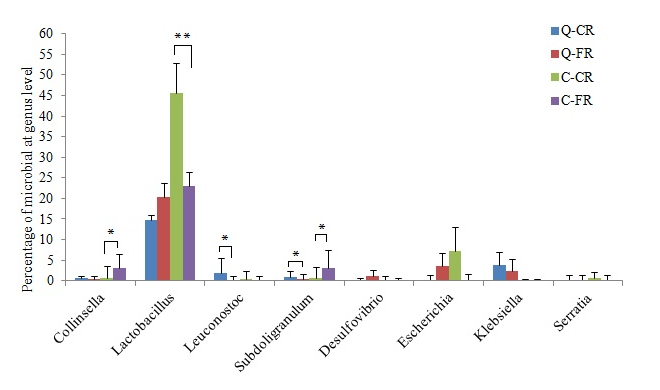


**Figure.S4**
